# Carbon prospecting in tropical forests for climate change mitigation

**DOI:** 10.1038/s41467-021-21560-2

**Published:** 2021-02-24

**Authors:** Lian Pin Koh, Yiwen Zeng, Tasya Vadya Sarira, Kelly Siman

**Affiliations:** 1 Centre for Nature-based Climate Solutions, and Department of Biological Sciences, National University of Singapore, 14 Science Drive 4, Singapore, 117543 Singapore; 2 School of Biological Sciences, The University of Adelaide, Adelaide, SA 5005 Australia

**Keywords:** Climate-change mitigation, Climate-change ecology

## Abstract

Carbon finance projects that protect tropical forests could support both nature conservation and climate change mitigation goals. Global demand for nature-based carbon credits is outpacing their supply, due partly to gaps in knowledge needed to inform and prioritize investment decisions. Here, we show that at current carbon market prices the protection of tropical forests can generate investible carbon amounting to 1.8 (±1.1) GtCO_2_e yr^−1^ globally. We further show that financially viable carbon projects could generate return-on-investment amounting to $46.0b y^−1^ in net present value (Asia-Pacific: $24.6b y^−1^; Americas: $19.1b y^−1^; Africa: $2.4b y^−1^). However, we also find that ~80% (1.24 billion ha) of forest carbon sites would be financially unviable for failing to break even over the project lifetime. From a conservation perspective, unless carbon prices increase in the future, it is imperative to implement other conservation interventions, in addition to carbon finance, to safeguard carbon stocks and biodiversity in vulnerable forests.

## Abstract (web summary)

Investing in forest protection is a way to generate tradable carbon credits to support biodiversity conservation and climate change mitigation. Here the authors assess and map the global supply of tropical forest carbon credits with the goal of informing climate policy and investments.

## Introduction

Nature-based solutions could contribute substantially to climate change mitigation^1,2^. These solutions include the protection, restoration, and improved management of forests, wetlands, grasslands, and agricultural lands to increase carbon dioxide sequestration, reduce emissions and enhance climate resilience^1,3^. Protecting and ensuring the health of natural ecosystems are also important for conserving biodiversity, providing clean air and water, safeguarding food security, and sustaining livelihoods^1,4^.

The climate mitigation potential and co-benefits of nature-based climate solutions, in turn, present exciting opportunities for the public and private sectors to meet their climate goals, invest in carbon finance, and contribute to addressing the impacts of climate change^5,6^. Indeed, with increasing support and interest from institutions such as the World Bank, the transacted volume of nature-based carbon credits in the voluntary carbon market grew by over 250% between 2016 and 2018, from 14 MtCO_2_e to 51 MtCO_2_e^6^.

The growing demand for high quality, nature-based carbon credits may be outpacing their supply^6,7^, as indicated by a 30% (or $1.1 t^−1^CO_2_e) increase in the average price of carbon offsets associated with nature-based solutions in 2019 compared to the previous year^7^. The bottleneck in shovel-ready carbon projects may be due in part to gaps in knowledge critical for supporting and informing investment decisions on both protecting existing carbon stocks (e.g., avoided deforestation) and enhancing new carbon stocks (e.g., reforestation). For example, while the protection of tropical forests could in principle contribute substantially to climate mitigation by safeguarding their forest carbon^8^, these carbon stocks may not all be fundable through carbon finance, and hence not all be investible.

In fact, only the subset of forest carbon stocks that are under imminent threat of decline or loss if left unprotected by a conservation intervention may be certifiable^9,10^. This criterion of ‘additionality’ is a pre-condition for certifying all carbon credits, including nature-based credits traded in the voluntary carbon market, under the rules of the United Nations Framework Convention on Climate Change and reducing emissions from deforestation and forest degradation (REDD) and REDD + programmes^9,11^.

**Fig. 1:**
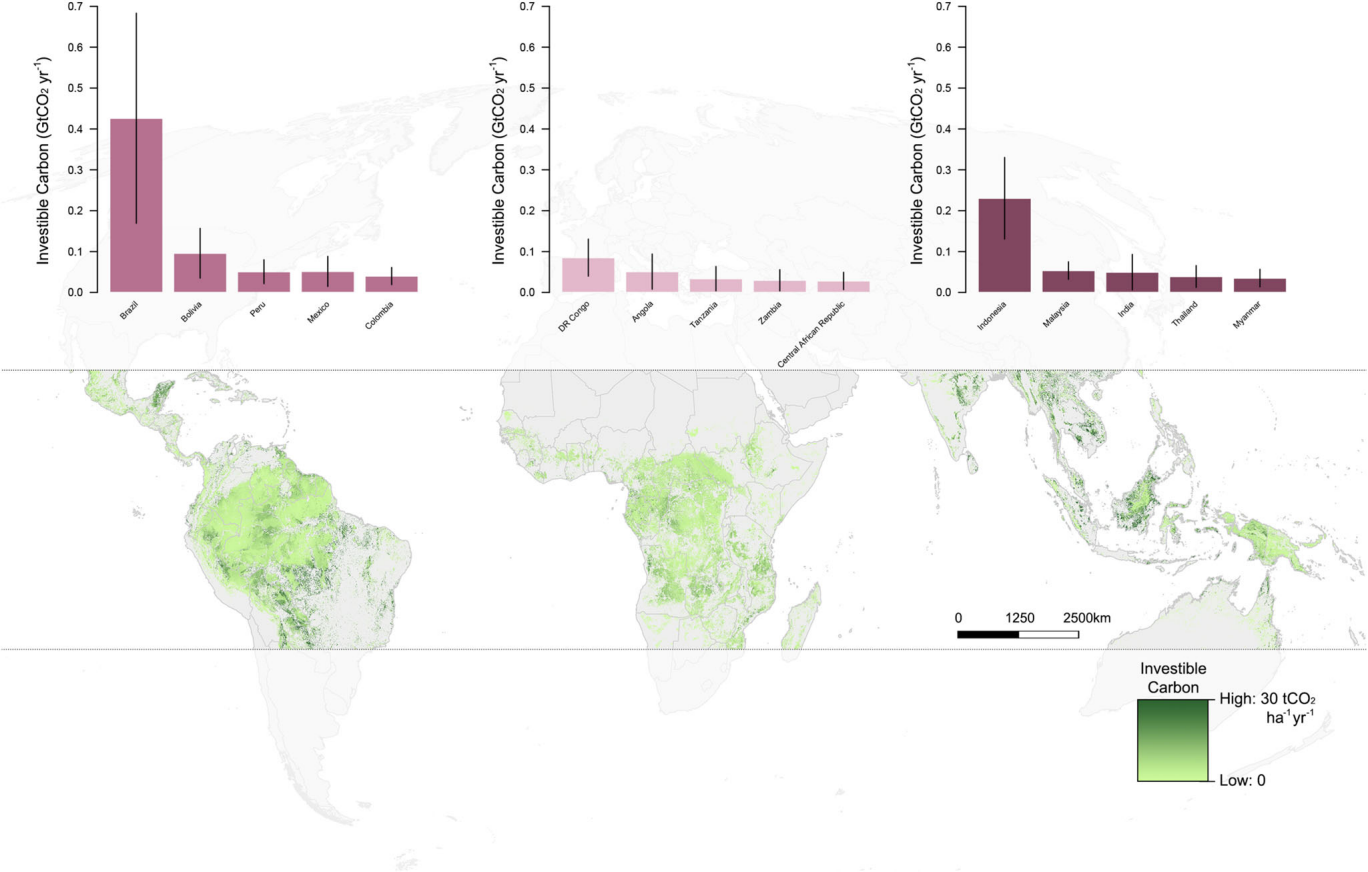
Global investible forest carbon across the tropics. The estimated volume of investible carbon from the five countries with the highest potential in each of the tropical regions of the Americas, Africa, and Asia-Pacific are highlighted.

We modeled the magnitude of certifiable carbon from forest protection projects (hereafter referred to as ‘investible forest carbon’), and its climate mitigation potential to produce a global investible forest carbon map at 1-km resolution (Fig. 1). We achieved this by analyzing the spatial distribution of existing forest carbon stocks^12^, with respect to their projected future risk of deforestation to account for additionality^13^, while also incorporating other key criteria of the Voluntary Carbon Standard (VCS)^9^ (see Methods for details).

## Results and discussion

### Investible forest carbon

Our analysis shows that the protection of tropical forests worldwide could generate investible carbon amounting to 1.8 (±1.1) GtCO2e yr^−1^ (Fig. 1; Table 1). Much of this carbon would originate from the Americas (809.1 ± 487.7 MtCO_2_ yr^−1^) and the Asia-Pacific region (581.8 ± 311.8 MtCO_2_ yr^−1^). The African continent has substantially lower potential for generating investible carbon from tropical forest projects (392.7 ± 286.5 MtCO_2_ yr^−1^). These findings are important to both potential investors of new forest protection carbon projects and local forest stakeholders for highlighting opportunities to generate revenue from carbon projects as an alternative to the business-as-usual scenario of destroying these forests.

**Table 1:** Global, regional, and country-level estimates of investible carbon and return-on-investment (based on net present value).

Region	Country	Investible carbon (tCO_2_ yr^−1^)	Net present value (USD y^−1^)
Global (Pantropic)		1,783,585,000 ± 1,086,018,000	46,032,396,000 ± 29,096,730,000
Americas		809,093,000 ± 487,743,000	19,057,800,000 ± 12,002,354,000
	Brazil	426,173,000 ± 256,966,000	11,207,547,000 ± 7,015,052,000
	Bolivia	95,975,000 ± 60,827,000	2,487,060,000 ± 1,662,773,000
	Peru	51,487,000 ± 36,756,000	1,200,075,000 ± 888,182,000
	Mexico	50,817,000 ± 29,152,000	616,169,000 ± 386,136,000
	Colombia	40,162,000 ± 21,079,000	692,353,000 ± 379,808,000
	Venezuela	35,649,000 ± 20,640,000	805,333,000 ± 485,447,000
	Paraguay	25,039,000 ± 19,983,000	860,262,000 ± 706,664,000
	Guyana	18,078,000 ± 6,309,000	143,976,000 ± 57,235,000
	Ecuador	12,843,000 ± 6,671,000	305,660,000 ± 166,689,000
	Suriname	12,206,000 ± 3,947,000	102,757,000 ± 37,687,000
	Cuba	5,773,000 ± 4,017,000	77,950,000 ± 59,825,000
	Guatemala	5,643,000 ± 4,843,000	190,663,000 ± 168,034,000
	Argentina	5,453,000 ± 3,363,000	96,907,000 ± 64,996,000
	French Guiana	4,414,000 ± 1,314,000	18,503,000 ± 6,300,000
	Nicaragua	4,356,000 ± 2,613,000	41,472,000 ± 27,713,000
	Honduras	3,758,000 ± 2,300,000	44,258,000 ± 29,349,000
	Panama	2,908,000 ± 1,790,000	21,525,000 ± 14,861,000
	Belize	2,429,000 ± 1,371,000	50,709,000 ± 30,777,000
	Dominican Republic	2,224,000 ± 1,475,000	50,888,000 ± 35,840,000
	Costa Rica	1,512,000 ± 868,000	5,869,000 ± 3,824,000
	El Salvador	721,000 ± 484,000	11,890,000 ± 8,471,000
	Jamaica	458,000 ± 268,000	6,493,000 ± 4,182,000
	Trinidad and Tobago	299,000 ± 181,000	3,764,000 ± 2,521,000
	Haiti	268,000 ± 205,000	3,915,000 ± 3,214,000
	Bahamas	235,000 ± 175,000	8,516,000 ± 6,551,000
	Guadeloupe	45,000 ± 30,000	538,000 ± 388,000
	Puerto Rico	35,000 ± 22,000	875,000 ± 579,000
	Cayman Islands	24,000 ± 15,000	177,000 ± 132,000
	Turks and Caicos Islands	24,000 ± 19,000	533,000 ± 452,000
	Antigua and Barbuda	23,000 ± 19,000	627,000 ± 522,000
	Saint Lucia	21,000 ± 13,000	222,000 ± 147,000
	Martinique	13,000 ± 8,000	147,000 ± 97,000
	Grenada	9,000 ± 5,000	11,000 ± 8,000
	Saint Kitts and Nevis	6,000 ± 4,000	64,000 ± 46,000
	Curaçao	4,000 ± 3,000	8,000 ± 9,000
	Saint Vincent and the Grenadines	3,000 ± 2,000	5,000 ± 3,000
	Montserrat	3,000 ± 2,000	26,000 ± 20,000
	Virgin Islands, U.S.	2,000 ± 1,000	37,000 ± 27,000
	Bonaire, Sint Eustatius and Saba	1,000 ± 1,000	6,000 ± 6,000
	Virgin Islands, British	1,000 ± 0	5,000 ± 4,000
Africa		392,659,000 ± 286,485,000	2,355,924,000 ± 1,859,685,000
	DR Congo	85,258,000 ± 45,293,000	295,462,000 ± 179,865,000
	Angola	51,008,000 ± 42,975,000	331,371,000 ± 302,712,000
	Tanzania	33,717,000 ± 29,792,000	235,594,000 ± 226,691,000
	Zambia	29,791,000 ± 25,982,000	169,617,000 ± 163,821,000
	Central African Republic	28,178,000 ± 21,167,000	41,380,000 ± 35,094,000
	Mozambique	27,671,000 ± 24,103,000	179,078,000 ± 169,465,000
	Congo	22,461,000 ± 11,264,000	198,163,000 ± 109,618,000
	Cameroon	20,546,000 ± 12,299,000	277,689,000 ± 172,424,000
	Gabon	18,982,000 ± 6,678,000	198,918,000 ± 75,359,000
	South Sudan	11,576,000 ± 11,390,000	8,147,000 ± 8,056,000
	Ethiopia	10,328,000 ± 9,863,000	80,770,000 ± 80,214,000
	Nigeria	8,291,000 ± 7,758,000	46,809,000 ± 47,219,000
	Madagascar	6,352,000 ± 5,271,000	30,719,000 ± 27,526,000
	Guinea	4,465,000 ± 3,965,000	21,594,000 ± 20,546,000
	Côte d’Ivoire	4,244,000 ± 3,385,000	21,897,000 ± 18,579,000
	Liberia	4,150,000 ± 4,143,000	2,479,000 ± 2,793,000
	Zimbabwe	3,942,000 ± 3,366,000	31,271,000 ± 27,839,000
	Ghana	3,442,000 ± 1,384,000	77,671,000 ± 32,957,000
	Kenya	2,361,000 ± 2,333,000	25,290,000 ± 26,105,000
	Equatorial Guinea	1,935,000 ± 1,947,000	3,818,000 ± 4,222,000
	Senegal	1,711,000 ± 1,505,000	3,052,000 ± 2,894,000
	Uganda	1,711,000 ± 1,737,000	6,234,000 ± 7,022,000
	Malawi	1,685,000 ± 535,000	36,083,000 ± 12,147,000
	Chad	1,552,000 ± 1,430,000	8,496,000 ± 8,446,000
	Benin	1,482,000 ± 1,473,000	3,887,000 ± 4,376,000
	Mali	960,000 ± 977,000	539,000 ± 724,000
	Guinea-Bissau	914,000 ± 926,000	69,000 ± 82,000
	Togo	901,000 ± 802,000	3,895,000 ± 3,853,000
	Sudan	816,000 ± 787,000	598,000 ± 665,000
	Sierra Leone	663,000 ± 430,000	9,949,000 ± 6,751,000
	Botswana	356,000 ± 360,000	1,827,000 ± 1,830,000
	South Africa	253,000 ± 245,000	1,211,000 ± 1,266,000
	Namibia	225,000 ± 225,000	108,000 ± 130,000
	Somalia	211,000 ± 216,000	22,000 ± 29,000
	Burkina Faso	201,000 ± 205,000	51,000 ± 60,000
	Burundi	135,000 ± 120,000	282,000 ± 261,000
	Gambia	86,000 ± 85,000	873,000 ± 900,000
	Rwanda	52,000 ± 45,000	278,000 ± 241,000
	Sao Tome and Principe	36,000 ± 17,000	723,000 ± 369,000
	Comoros	6,000 ± 4,000	2,000 ± 2,000
	Mayotte	6,000 ± 3,000	13,000 ± 9,000
Asia-Pacific		581,832,000 ± 311,790,000	24,618,672,000 ± 13,578,652,000
	Indonesia	230,478,000 ± 99,746,000	10,126,046,000 ± 4,508,720,000
	Malaysia	53,632,000 ± 21,367,000	2,598,219,000 ± 1,067,413,000
	India	49,742,000 ± 43,363,000	1,734,079,000 ± 1,560,695,000
	Thailand	39,054,000 ± 26,658,000	1,741,889,000 ± 1,217,483,000
	Myanmar	35,182,000 ± 21,480,000	1,239,052,000 ± 784,774,000
	Australia	33,746,000 ± 23,335,000	1,357,093,000 ± 968,389,000
	Cambodia	28,307,000 ± 17,179,000	1,479,793,000 ± 915,994,000
	China	28,294,000 ± 16,090,000	1,327,222,000 ± 774,169,000
	Viet Nam	24,031,000 ± 14,475,000	1,111,770,000 ± 686,509,000
	Laos	22,123,000 ± 10,166,000	932,369,000 ± 444,216,000
	Papua New Guinea	16,504,000 ± 6,223,000	139,478,000 ± 59,378,000
	Philippines	10,133,000 ± 5,156,000	346,747,000 ± 184,460,000
	Sri Lanka	4,154,000 ± 2,934,000	208,398,000 ± 149,681,000
	Bangladesh	4,154,000 ± 2,541,000	192,832,000 ± 121,009,000
	Brunei Darussalam	1,101,000 ± 432,000	43,305,000 ± 17,682,000
	Taiwan	598,000 ± 308,000	17,154,000 ± 9,304,000
	Timor-Leste	471,000 ± 261,000	18,894,000 ± 10,867,000
	Hong Kong	125,000 ± 73,000	4,269,000 ± 2,628,000
	Singapore	1,000 ± 1,000	62,000 ± 53,000

Uncertainties indicated are based on standard deviation.

Our estimates of investible forest carbon are ~31% lower than those reported in previous studies^1,3^, which were largely based on aggregated country level data on carbon stocks and deforestation rates. Furthermore, our analysis also incorporates VCS criteria, such as the requirement to set aside buffer credits^9^, not considered in other studies^1,3^. As such, we are able to compare our estimates of investible forest carbon with actual volumes of verified carbon units (VCUs) reported by 25 real-world VCS projects on forest protection across the tropics. We achieved that based on empirical VCS data, including 111 ground-based and verified carbon stock measurements from projects across 16 countries. We find a relatively strong correlation (*R* = 0.49, *p* < 0.05) and no significant difference (*t* = 0.92, *p* = 0.36) between our estimates and the ground-based measurements of verified carbon units (Fig. S1).

Globally, the top five countries in terms of investible carbon are Brazil (426.2 ± 257.0 MtCO_2_ yr^−1^), Indonesia (230.5 ± 99.7 MtCO_2_ yr^−1^), Bolivia (96.0 ± 60.8 MtCO_2_ yr^−1^), Democratic Republic of Congo (85.3 ± 45.3 MtCO_2_ yr^−1^) and Malaysia (53.6 ± 21.4 MtCO_2_ yr^−1^) (Fig. 1; Table 1).

Barriers to the establishment of forest carbon projects may include competing interests and priorities from other economic sectors (e.g., agriculture), lack of enabling conditions and policies, governance and institutional constraints, and prohibitively high technical entry bar^14–16^. Such barriers can further limit the potential of forest protection as a nature-based climate solution when compared to other forestry and forest restoration based techniques^17,18^. Many of these barriers may be overcome if actionable information regarding both the financial risks and return-on-investment of projects is available to incentivize solutions.

### Financially viable forest carbon

Indeed, investible carbon projects may not all be profitable. The financial viability of a project depends on a range of factors, including operational costs and carbon pricing, as well as political risk, which may vary with location and over time^19^.

**Fig. 2:**
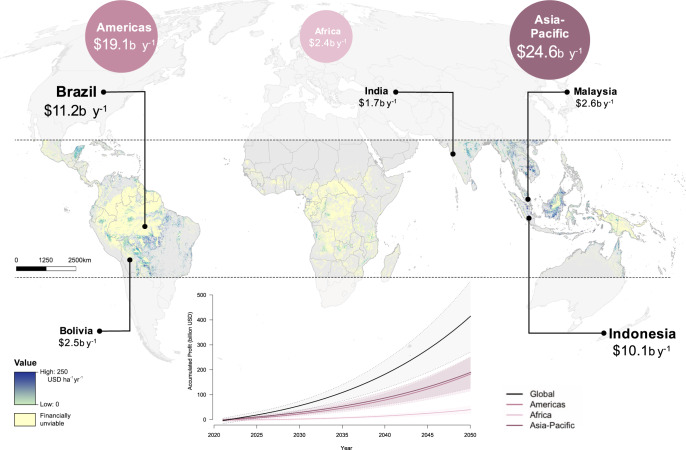
Global forest carbon return-on-investment from financially viable sites. Estimates are presented as net present values over a 30-year timeframe, highlighting the corresponding estimates across each region and five countries with the highest potential. We also present the accumulation of profits overtime at the global and regional levels, with shadings around the lines representing standard deviation (inset).

We modeled the relative profitability of these projects to produce a global forest carbon return-on-investment map (Fig. 2).

We based our analysis on several simplifying assumptions (see Methods for details). Briefly, we applied a cost estimate of $25 ha^−1^ for project establishment, and $10 ha^−1^ y^−1^ for subsequent years for project maintenance. We also assumed a constant carbon price of $5.8 t^−1^CO_2_ for the first five years, followed by a 5% price appreciation for the subsequent years over a project timeframe of 30 years^19^. Finally, we applied a risk-adjusted discount rate of 10% in our calculation of net present values (NPV) for the return-on-investment of tropical forest carbon projects^20,21^.

We find that the vast majority of financially viable (i.e., yielding positive NPV) and most profitable forest carbon sites (>$308 ha^−1^ y^−1^; 90th percentile) are located in the Asia-Pacific region with NPV amounting to $24.6b y^−1^, compared to the Americas ($19.1b y^−1^) and Africa ($2.4b y^−1^) (Fig. 2 & S2; Table 1). This largely reflects the fact that tropical forests in the Asia-Pacific region both contain high carbon density and are facing high deforestation risks, thereby creating immense opportunities for avoiding carbon emissions through forest protection^22^. Of course, the high risk of deforestation may also pose a challenge to the long-term environmental integrity of these projects, which also needs to be considered and mitigated through other national or region-specific policy measures.

The top five countries with highest return-on-investment are Brazil ($11.2b y^−1^), Indonesia ($10.1b y^−1^), Malaysia ($2.6b y^−1^), Bolivia ($2.5b y^−1^) and India ($1.7b y^−1^) (Fig. 2 & S2; Table 1). This represents a substantial amount of potential returns from the trading of carbon credits in markets that could support the protection of forests, thereby benefiting forest stakeholders.

Globally, ~80% (1.24 billion ha) of the investible forest carbon sites would be financially unviable for carbon finance for failing to break even over the project lifetime (i.e., yielding negative NPV; Fig. 2). Importantly, these forests represent forgone climate mitigation at a rate of 0.7 GtCO_2_ yr^−1^, assuming no other conservation measures are taken. From a forest conservation perspective, these findings suggest that carbon finance will fail to protect the vast majority of investible carbon sites, which are also, by definition, vulnerable to deforestation (Fig. 2 & S2; Table 1).

**Fig. 3:**
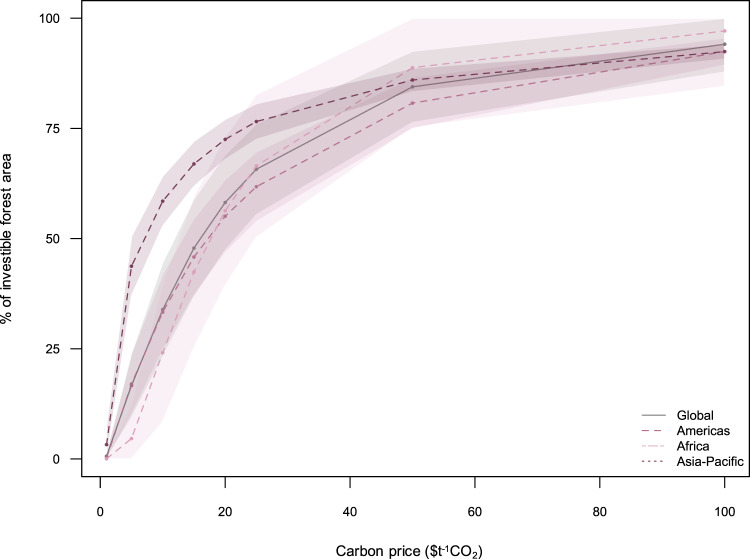
Effect of carbon pricing on the financial viability of forest carbon sites. Graph indicates the proportion of investible forest carbon that are financially viable for carbon finance. Shadings around the lines represent confidence bands based on standard deviation.

However, if global demands for nature-based carbon credits continue to grow^6^, future carbon prices may also increase. We modeled the effects of carbon pricing on the financial viability of forest carbon sites globally. We find that at a global level, carbon pricing at $16 t^−1^CO_2_ and $44 t^−1^CO_2_ are needed to protect 50 and 80% of investible carbon sites, respectively (Fig. 3). Further carbon price increases above $50 t^−1^CO_2_ would only bring marginal forest conservation and climate mitigation benefits (Fig. 3). We also find that this sensitivity to carbon pricing varies geographically. For example, carbon prices of $7.1 t^−1^CO_2_ for Asia-Pacific, $17.2 t^−1^CO_2_ for the Americas, and $17.7 t^−1^CO_2_ for Africa, are needed to protect 50% of the investible forest carbon sites in these respective regions (Fig. 3). These carbon prices may be potentially achievable in the near future, given the growing global interest in nature-based carbon credits, and suggests that carbon pricing may be an important lever to drive investments for nature-based climate solutions through carbon finance^6,7^.

However, in the same way that carbon pricing can affect the financial viability of forest carbon projects, so too can changes in the operating expenses of these projects. In a separate analysis, we find that increases in the establishment and annual maintenance costs by 50% and 100% would result in decreases in the global volumes of profitable forest carbon by 19.6% to 892.1 ± 559.2 MtCO_2_ yr^−1^ and 34.3% to 728.8 ± 462.2 MtCO_2_ yr^−1^, respectively (see Methods; Table S1).

Furthermore, it is also important to note that some financially viable but less profitable forest carbon sites will struggle to compete with lucrative land uses, particularly in countries such as Brazil and Indonesia, which are the world’s major producers of soy, beef, and palm oil^19^. In other countries, such as the Democratic Republic of the Congo, hydrocarbon exploration and logging developments with multiple vested interests may pose additional barriers to carbon projects^23^.

To understand the potential impacts of these opportunity costs, we performed another separate analysis that takes into account the land rent from potential agricultural and forestry developments that may compete with forest carbon projects (see Methods). We find when all forests are excluded where opportunity costs are higher than returns from carbon finance, the global climate mitigation potential of forest protection would decrease by 47.7% to 580.6 ± 368.6 MtCO_2_ yr^−1^ (Table S1). Therefore, it is imperative to implement other conservation strategies and interventions to safeguard the carbon stocks and biodiversity in these vulnerable forests. Critically, carbon finance must be placed in the broader context of other incentives and policies targeting both the corporate and government sectors to protect forests and avoid emissions. An example is Costa Rica’s model of using part of their carbon tax revenue to support the protection and restoration of natural ecosystems across the country, with direct payments made to farmers and landowners for their compliance^24^.

Obviously, there is a wide range of environmental, socioeconomic, governance, and geopolitical factors that can influence climate strategies, conservation actions, and investment decisions^25^. For example, some carbon projects may include financially unviable sites that are important for conserving biodiversity, safeguarding rural livelihoods, or providing other co-benefits that may be highly valued by society but not internalized in our analysis^4^. Such financially unviable sites could potentially become viable by leveraging on payment schemes for other ecosystem services beyond carbon storage.

Furthermore, the political ecology landscape of existing and new carbon investments within a host country may also influence and alter the risk of deforestation, which affects additionality, the long-term success of forest carbon projects, and ultimately the permanence of carbon credits or the diversion of development and deforestation to other locations (i.e., leakage effects)^10,11^. For example, the political risk for certified carbon credits has recently increased significantly in Brazil. In exchange for political support, the Brazilian government laid the foundation for landowners to accelerate deforestation^26^. This political bargaining may have seriously compromised Brazil’s ability to meet the Paris target. These political risk considerations are crucial to ensure the long-term viability of carbon investments.

One approach to mitigate the risks of non-permanence and leakage effects of forest carbon projects is to require project developers to set aside buffer credits, which is in fact already a requirement under the VCS (i.e., requiring 20% of total credits to be set aside as buffer). We find that if this requirement is increased by an additional 10%, 20%, or 30% will decrease the global volume of profitable forest carbon by 18.1% to 909.1 ± 567.4 MtCO_2_ yr^−1^, 35.4% to 716.7 ± 448.4 MtCO_2_ yr^−1^ or 51.7% to 535.8 ± 336.3 MtCO_2_ yr^−1^, respectively (see Methods; Table S1).

Our analyses draw from a sliver of the best available data to provide a snapshot of the relative investible carbon and return-on-investment for the protection of tropical forests as a nature-based climate solution. While we find that carbon finance may fail to protect a large proportion of tropical forests at current carbon prices, nature-based climate solutions remain hugely important for the many other co-benefits they provide for society. By clarifying some of these opportunities and constraints, we help to calibrate expectations and incentivize public and private sector investments in nature-based climate solutions to benefit the environment, climate, and society.

## Methods

### Overview of methods

First, we modeled and mapped investible forest carbon, and its climate mitigation potential across the tropics at 1-km resolution. Second, we compared our estimates of investible forest carbon with actual volumes of VCUs reported by 25 real-world VCS forest protection projects. Third, we modeled the relative profitability of investible forest carbon sites to produce a global forest carbon return-on-investment map based on their NPV.

All calculations were based on data dated between 2012 and 2017 and at a resolution of 0.00833 degrees (~1 km). To ensure data standardization, we resampled (bilinear) finer-scaled data where necessary, for example, for data sourced from the European Space Agency - Climate Change Initiative -Land Cover^27^. We only considered tropical forests between ~23.44°N and 23.44°S, and excluded all land cover types that would preclude forests, for example, savannas, bare ground, water, agriculture and urban areas^27^.

### Investible forest carbon

We first estimated the total volume of CO_2_ associated with three carbon pools in tropical forests: aboveground carbon, belowground carbon, and soil organic carbon. Next, we applied key VCS criteria, including additionality, to model and map investible forest carbon across the tropics.

### Mapping total volume of CO_2_ associated with tropical forests

#### Aboveground carbon

We applied a stoichiometric factor of 0.475 to recent spatial data on aboveground carbon biomass^12^ (i.e., for period 2012–2016), to convert it from biomass to carbon stock values, based on established carbon accounting methodology^3,28,29^. We performed an uncertainty analysis to account for potential variability in this stoichiometric factor (see ‘Uncertainty analysis’ section below). We applied a conversion factor of 3.67 to derive the volume of CO_2_ associated with this carbon pool^3^.

#### Belowground carbon

We derived belowground carbon biomass by applying two different allometric equations relating root to shoot biomass^30^ to the most recent spatial dataset on aboveground carbon biomass^12^, following established carbon accounting methodology^3,28,29^. The two equations are: belowground biomass = 0.489 × aboveground biomass^0.89; and belowground biomass = 0.26 × aboveground biomass. We then applied a stoichiometric factor of 0.475 to the estimated belowground carbon biomass to convert it from biomass to carbon stock values. Next, we calculated the mean, minimum and maximum values for belowground carbon based on an uncertainty analysis (see ‘Uncertainty analysis’ section below). We applied a conversion factor of 3.67 to derive the volume of CO_2_ associated with this carbon pool^3^.

#### Soil organic carbon

We also considered soil carbon due to its potentially significant contributions to carbon storage^31^ and despite potential uncertainties and variability surrounding its measurements^32^. Specifically, we utilized the organic carbon density of the topsoil layer (0–30 cm) obtained from the European Soil Data Centre^33^ as it represented the best data available of soil organic carbon. We applied a conversion factor of 3.67 to derive the volume of CO_2_ associated with this carbon pool^3^.

### Applying VCS criteria to map investible forest carbon

The criterion of additionality is a pre-condition for certifying all carbon credits under the VCS. This implies that only the volume of forest carbon that are under imminent threat of decline or loss if left unprotected by a conservation intervention can be certified under the VCS. We derived the volume of forest carbon under threat of loss based on best available proxy data on projected future deforestation rates across the tropics^13^ (through to the year 2029), and annualized over the prediction period (15 years). We applied this estimated annual deforestation rate to the total volume of CO_2_ associated with tropical forests as estimated above, to derive the volume of CO_2_ that would be certifiable and therefore investible under the VCS.

We also assumed a conservative 10-year decay estimate for the belowground carbon pool^9^.

Additionally, we excluded lands that will likely not be certifiable for other reasons^9^, including recently deforested areas^34^ (i.e., for the period 2010–2017), as well as human settlements located within these forests^35^.

Lastly, we accounted for the VCS requirement to set aside buffer credits of 20% to account for the risk of non-permanence associated with Agriculture, Forestry and Other Land Use projects (AFOLU)^9^.

### Comparing estimates of investible forest carbon to verified carbon units

We compared our estimates of investible forest carbon with actual volumes of VCUs reported by real-world VCS forest protection projects (https://verra.org/).

We identified a set of 25 VCS forest protection projects from across 16 countries that met the following criteria: ii) includes spatial data on project boundary in their project documentation; ii) the project extent is located entirely within the tropics; and 3) has been verified (i.e., either “verified, under verification” or “verification approve”) (Table S2).

We extracted the shapefiles (i.e., geometric polygons) of these VCS projects, and overlay them on our map of investible forest carbon to extract the volume of investible forest carbon (CO_2_) from our analysis that corresponds to each of the 25 VCS forest project.

We then compared our estimates of investible forest carbon to the volume of VCUs issued between 2005 and 2018 for each VCS project. The number of data points reported per year for each project ranged from 1–10, and generated a total of 111 data points for comparison. We then assessed the degree of correlation (i.e., Pearson’s correlation), relative accuracy (i.e., Root Mean Square Error; RMSE), and statistical difference (i.e., paired t-test) between the two datasets.

### Estimating return-on-investment

Based on our map of investible forest carbon, we modeled the relative profitability of investible forest carbon sites to produce a global forest carbon return-on-investment map based on their NPV. We calculated NPV of these returns based on several simplifying assumptions following established values from previous studies^19^.

First, we estimated the cost of project establishment at $25 ha^−1^. This was based on a wide range of costs that are key to the development of a project, including but not limited to project design, governance and planning, enforcement, zonation, land tenure and acquisition, surveying and research^19,36,37^.

Second, we estimated an annual maintenance cost of $10 ha^−1^, which included aspects such as education and communication, monitoring, sustainable livelihoods, marketing, finance and administration^19,36,37^.

Third, we assumed a constant carbon price of $5.8 t^−1^CO_2_ for the first five years. This price was based on an average price of carbon for avoided deforestation projects recently reported by Forest Trends’ Ecosystem Marketplace^6^ (i.e., for the period 2006–2018). After the first five years, we assumed a 5% price appreciation for subsequent years over a project timeframe of 30 years^19^.

Based on these criteria, we calculated NPV of annual and accumulated profits over the 30 years, based on a 10% risk-adjusted discount rate.

Separately, we repeated the analysis using a range of starting carbon prices, including $1, $5, $10, $15, $25, $50, $100 t^−1^CO_2_, based on cost effectiveness thresholds from previous studies^1^. In these analyses, other assumptions remain unchanged, including the project establishment and annual maintenance cost, price appreciation, discount rates and timeframe. Based on these criteria and excluding sites that would be unable to breakeven (i.e., yielding net negative NPVs), we calculated the potential profitable forest areas, as a percentage of the total investible forest areas, associated with these different starting carbon prices.

All values of investible carbon and return-on-investment (based on NPV) were summarized to global, regional, and country level estimates (see Table 1). For countries that extend beyond tropical latitudes, we only analyze and present data for their tropical extents. These values were rounded to the nearest 1000 values.

### Uncertainty analyses

#### Stoichiometric factor

Previous studies utilized a range of stoichiometric factors, typically ranging between 0.45 and 0.50^3,28,29^. We account for this variability by first using a stoichiometric factor of 0.475, which was based on the median value across these reference studies^3,28,29^. We then repeated the analyses with stoichiometric factors of 0.45 and 0.50 to calculate the respective minimum and maximum values of above and belowground carbon per cell.

#### Root to shoot biomass allometric equations

Many site-specific factors can influence the ratio of root to shoot biomass, resulting in variability of the best-fit allometric equations^30^. Here, we account for this variability by utilizing the two allometric equations that best matches global data^30^. This produced two sets of spatially explicit estimates of belowground biomass, from which we calculated the average, minimum and maximum values per cell.

#### Aboveground biomass

We incorporated uncertainties, reported at standard deviations, which were inherent to the aboveground biomass dataset^12^.

#### Leakage effects

We considered three scenarios of leakage, where the protection of an area of forest results in deforestation beyond its borders to the amounts of 10%, 20%, and 30% of the areas’ carbon volume. This reduces the total investible carbon within each cell, thereby causing a decrease in return-on-investment and the climate mitigation potential within profitable areas to 81.9 ± 51.1, 64.6 ± 40.4 and 48.3 ± 30.3%, or 909.1 ± 567.4, 716.7 ± 448.4, 535.8 ± 336.3 MtCO_2_ yr^−1^, respectively (Table S1).

#### Establishment and maintenance costs

We also considered two scenarios of establishment and maintenance cost, where the overall direct cost of protecting areas from deforestation increases by 50% and 100%. We find that this reduces the climate mitigation potential in profitable areas to 80.4 ± 50.4 and 65.7 ± 41.6% or 892.1 ± 559.2 and 728.8 ± 462.2 MtCO_2_ yr^−1^ respectively (Table S1).

#### Opportunity costs

We also considered the potential for alternative land-use such as agriculture or timber extraction to outcompete the value of protecting forests through carbon financing means. Utilizing agricultural rents (based on 18 crops) and timber value as a proxy for opportunity cost^38^, we excluded areas where opportunity cost exceeds projected net present values. This results in a large decrease in overall climate mitigation potential, almost comparable to the 30% leakage scenario, to 52.3 ± 33.2% or 580.6 ± 368.6 MtCO_2_ yr^−1^ within remaining areas.

All analyses were performed in R version 3.6.0^39^, utilizing the package “raster” for processing and calculations of raster layers^40^. Map visualizations were formed in QGIS^41^.

### Reporting summary

Further information on research design is available in the Nature Research Reporting Summary linked to this article.

## Supplementary information

Supplementary Information

Reporting Summary

## Data Availability

Validation data are summarized in Table S1. All maps generated are available from Zenodo, 10.5281/zenodo.4287780

## Code availability

All R scripts used to generate maps are available from Zenodo, 10.5281/zenodo.4287780
