# Supplementary material for: Carbon prospecting in tropical forests for climate change mitigation
Source: Nat Commun. 2021 Feb 24;12:1271. doi: 10.1038/s41467-021-21560-2 (PMC7904812; doi:10.1038/s41467-021-21560-2)
Supplement: Supplementary file 1 — Supplementary Information [file 41467_2021_21560_MOESM1_ESM.pdf]

Carbon prospecting in tropical forests for climate change mitigation

Lian Pin Koh[1], Yiwen Zeng[2], Tasya Vadya Sarira, Kelly Siman

Correspondence to: [1]lianpinkoh@nus.edu.sg; [2]zengyiwen@nus.edu.sg

This PDF file includes:

Supplementary Tables 1 to 2

Supplementary Figure 1 to 2

Supplementary Table 1: Sensitivity analyses measuring the change in climate mitigation potential (CMP) associated with profitable areas. These account for leakage (10%, 20% and 30%), increase in development and maintenance cost (50% and 100%), and opportunity cost (from agricultural rents and timber production).

Country	Original analysis (CMP)	Leakage: 10% (% CMP)	Leakage: 20% (% CMP)	Leakage: 30% (% CMP)	Cost: +50% (% CMP)	Cost: +100% (% CMP)	Opportunity Cost (% CMP)
**World**	**1,783,584,800 ±1,086,018,100**	**81.9 ±51.1**	**64.6 ±40.4**	**48.3 ±30.3**	**80.4 ±50.4**	**65.7 ±41.6**	**52.3 ±33.2**
**Americas**	**809,093,200 ±487,743,200**	**81 ±50.3**	**62.7 ±39**	**45.4 ±28.4**	**76.5 ±47.8**	**58.6 ±37.2**	**59.5 ±37.2**
Brazil	426,173,000 ±256,966,200	82.1 ±50.7	65.3 ±40.3	49.5 ±30.7	82 ±50.7	68.5 ±42.9	62.8 ±39.3
Bolivia	95,975,000 ±60,827,300	80.5 ±52.8	60.8 ±40.2	42 ±28.1	72.3 ±48.1	48.7 ±33.5	77.8 ±51.1
Mexico	51,486,600 ±36,756,100	81.7 ±59.3	63.1 ±46.1	43.8 ±32.4	75.6 ±55.6	40.9 ±32.5	49.1 ±35.9
Peru	50,817,100 ±29,151,800	74.6 ±45.7	47.8 ±30.6	24.1 ±15.7	46.4 ±30.3	25 ±15.6	3.8 ±2.5
Colombia	40,161,800 ±21,079,200	81.9 ±44	64.2 ±34.7	47.1 ±25.6	79.1 ±42.9	61.3 ±34	33 ±18.5
Venezuela	35,649,200 ±20,640,000	82.2 ±48.7	64.3 ±38.3	47.3 ±28.4	79.5 ±47.4	62.4 ±37.7	62.1 ±36.7
Paraguay	25,038,700 ±19,983,200	82.4 ±66.9	64.9 ±52.9	49.5 ±40.2	81.4 ±66.2	73.8 ±59.7	98.6 ±79.9
Guyana	18,077,700 ±6,308,500	70.2 ±27.8	46.8 ±18.3	29 ±11.5	51.6 ±20.3	28.9 ±11.8	18.3 ±7.6
Ecuador	12,842,900 ±6,670,800	82.9 ±44	66 ±35.2	48.7 ±26.4	81.8 ±44.1	62.3 ±34.6	47.5 ±25.8
Suriname	12,206,400 ±3,946,600	75.5 ±26.3	53.1 ±19.3	28.6 ±11.2	54.5 ±21.3	25.4 ±9.9	14.9 ±5.3
Cuba	5,772,900 ±4,017,400	70.6 ±52.9	41.9 ±32.3	17.9 ±14.6	37.6 ±30.4	8.5 ±7.5	85.8 ±62.5
Argentina	5,642,700 ±4,843,300	84.6 ±73.5	69.7 ±60.6	55 ±48	90 ±78.4	79.9 ±70.2	34.8 ±30.3
Guatemala	5,453,000 ±3,363,300	79 ±50.8	55.8 ±37.3	28.1 ±20.3	56 ±39.7	17.9 ±13.5	13.1 ±9.1
French Guiana	4,413,500 ±1,314,000	77.7 ±24.9	56.1 ±18.9	34.3 ±12.1	61.5 ±21.1	29.1 ±11.2	2.2 ±0.8
Nicaragua	4,355,700 ±2,613,500	68.5 ±44.6	39.1 ±26.6	16 ±11.5	34 ±24	8.1 ±6	64.7 ±40.9
Honduras	3,757,700 ±2,300,100	73.1 ±47.5	48.9 ±32.2	27.9 ±19	51.6 ±34.6	22.2 ±15.7	44.3 ±29
Panama	2,907,800 ±1,790,400	66.9 ±45.4	39.1 ±27	18.4 ±13.1	36.7 ±25.9	12.1 ±9	23 ±15.4
Belize	2,429,400 ±1,370,500	78.7 ±46.5	55.4 ±33.6	33.5 ±20.8	60.7 ±37.3	33.4 ±20.9	14.6 ±9
Dominican Republic	2,224,200 ±1,475,300	79.2 ±54.4	57.2 ±40.1	35.7 ±25.4	63.5 ±45	34 ±25.2	37.2 ±25.5
Costa Rica	1,512,000 ±868,300	61.6 ±39.8	33.3 ±21.8	14.3 ±9.5	28.9 ±19.2	8.4 ±5.7	59.1 ±37.1
El Salvador	720,900 ±484,100	76.1 ±53.3	51.8 ±36.6	30.4 ±21.9	56.6 ±40.9	24.9 ±18.3	99.8 ±69
Jamaica	457,900 ±267,700	73.8 ±46.5	47.9 ±30.9	24.9 ±16.7	47.6 ±31.3	17 ±11.8	100 ±61.7
Trinidad and Tobago	299,300 ±180,600	66.5 ±44.1	39.5 ±26	20.5 ±14	39.5 ±26.8	15.9 ±10.9	30.7 ±19

Supplementary Table 1 (con't): Sensitivity analyses measuring the change in climate mitigation potential (CMP) associated with profitable areas. These account for leakage (10%, 20% and 30%), increase in development and maintenance cost (50% and 100%), and opportunity cost (from agricultural rents and timber production).

Country	Original analysis (CMP)	Leakage: 10% (% CMP)	Leakage: 20% (% CMP)	Leakage: 30% (% CMP)	Cost: +50% (% CMP)	Cost: +100% (% CMP)	Opportunity Cost (% CMP)
Haiti	268,500 ±204,800	74.1 ±59.7	49.1 ±39.7	26.2 ±22.1	51.1 ±42.5	15.6 ±14.2	27.6 ±21.6
Bahamas	234,900 ±174,900	83.6 ±63.2	66.7 ±50.7	49.7 ±38.3	83.6 ±64	63.6 ±50.4	100 ±75.3
Guadeloupe	44,800 ±29,700	68.9 ±49.3	45.2 ±32.7	24.2 ±17.9	44 ±31.1	13.1 ±11.9	100 ±70.9
Puerto Rico	34,800 ±21,700	82.7 ±52.7	60.7 ±40.3	37.8 ±24.8	63.1 ±42.4	41.5 ±28.6	100 ±63.4
Cayman Islands	24,200 ±15,100	52 ±38.1	25.5 ±21.9	5.5 ±4.2	13 ±12.3	6.4 ±4	100 ±72.1
Antigua and Barbuda	23,500 ±19,100	75.4 ±62.5	56.7 ±47.3	32.3 ±28	61 ±53.6	26.7 ±25.2	100 ±82.7
Turks and Caicos Islands	23,300 ±18,500	81 ±66	58 ±48.7	38.9 ±33.5	67.8 ±55.5	32.6 ±29.5	100 ±80.6
Saint Lucia	21,500 ±12,600	68.4 ±42.4	40.7 ±27	16.2 ±11.7	39.7 ±27.9	10.1 ±8.5	100 ±63.6
Martinique	13,400 ±8,200	79 ±51.4	45.1 ±29.5	14.7 ±12.3	40.9 ±30.8	4.5 ±4	100 ±61
Grenada	8,800 ±5,100	67.2 ±45	0 ±5.9	0 ±0	0 ±0	0 ±0	100 ±57.8
Saint Kitts and Nevis	6,400 ±4,400	61.4 ±46.2	46.6 ±32.1	38.9 ±26.7	62.2 ±42.8	49.7 ±34.2	100 ±72.6
Curaçao	3,600 ±3,400	68.5 ±76.3	0 ±6.4	0 ±0	0 ±0	0 ±0	100 ±119
Saint Vincent and the Grenadines	3,300 ±2,100	87.5 ±55.6	75 ±68.4	0 ±0	0 ±0	0 ±0	100 ±85
Montserrat	3,200 ±2,300	68.8 ±56.8	40.8 ±30.4	34 ±25.4	54.3 ±40.6	54.3 ±40.6	100 ±74.4
Virgin Islands, U.S.	1,800 ±1,200	87.5 ±62	57.1 ±41.3	20.8 ±19.8	48.8 ±41.5	0 ±0	100 ±67.6
Bonaire, Sint Eustatius and Saba	1,200 ±1,100	50.1 ±45.6	43 ±39	0 ±0	0 ±0	0 ±0	100 ±91.2
Virgin Islands, British	600 ±400	35 ±22.9	30 ±28	0 ±0	0 ±0	0 ±0	100 ±82.2
Barbados	200 ±200	87.5 ±71	0 ±22.5	0 ±0	0 ±0	0 ±0	100 ±81.2
**Africa**	**392,659,400 ±286,485,300**	**67.4 ±53.3**	**41.3 ±32.8**	**23.5 ±18.6**	**42.8 ±33.9**	**24.9 ±19.5**	**69.9 ±55.1**
DR Congo	85,257,800 ±45,293,000	60 ±37.6	33.5 ±20.3	18.8 ±11.2	34.3 ±20.4	19 ±11.2	39.3 ±24.3
Angola	51,008,400 ±42,975,100	66.8 ±60.5	40.3 ±36.9	21 ±19.4	40 ±36.8	16.8 ±16	99.1 ±89.1
Tanzania	33,716,700 ±29,792,000	64.8 ±61.6	36.1 ±35	16.4 ±16.2	32.9 ±32.3	10.4 ±10.6	97.8 ±91.8
Zambia	29,791,200 ±25,981,700	65.9 ±62.9	31.1 ±31.4	10.7 ±10.8	23.2 ±23.6	7 ±6.9	100 ±92.3
Central African Republic	28,178,200 ±21,167,400	60.9 ±51.5	31.9 ±27.3	14.1 ±12.2	28.4 ±24.5	10.7 ±9.1	96.4 ±80.5
Mozambique	27,670,900 ±24,102,500	67.1 ±62.7	39.8 ±37.9	20 ±19.4	38.7 ±37.1	15.4 ±15.2	100 ±92.5
Congo	22,461,100 ±11,264,400	67.4 ±37.7	41.5 ±23.2	25.7 ±13.8	44.7 ±24.3	32.1 ±17.2	22.2 ±12.4
Cameroon	20,545,600 ±12,299,300	79.5 ±49.4	61.8 ±38.1	47 ±28.8	77.5 ±47.6	68.6 ±42	21.7 ±14.2
Gabon	18,982,000 ±6,677,500	77.8 ±29.2	58.8 ±22	42.8 ±16	71.8 ±26.8	59.1 ±22.1	36.8 ±14.8

Supplementary Table 1 (con't): Sensitivity analyses measuring the change in climate mitigation potential (CMP) associated with profitable areas. These account for leakage (10%, 20% and 30%), increase in development and maintenance cost (50% and 100%), and opportunity cost (from agricultural rents and timber production).

Country	Original analysis (CMP)	Leakage: 10% (% CMP)	Leakage: 20% (% CMP)	Leakage: 30% (% CMP)	Cost: +50% (% CMP)	Cost: +100% (% CMP)	Opportunity Cost (% CMP)
South Sudan	11,575,800 ±11,390,500	76.3 ±74.7	41.7 ±41.5	9.4 ±9.7	25.6 ±26	0.8 ±0.9	100 ±98.7
Ethiopia	10,327,900 ±9,862,700	75.3 ±74.5	55.1 ±54.6	38.3 ±37.9	65.4 ±64.8	49.5 ±49	100 ±99.2
Nigeria	8,290,900 ±7,758,100	65.5 ±65.8	38.8 ±39.1	20.7 ±21.1	39 ±39.5	18.1 ±18.7	42 ±43
Madagascar	6,352,100 ±5,271,300	68 ±60.5	43 ±38.4	24 ±21.8	44.4 ±39.9	21.8 ±20.1	61.9 ±55
Guinea	4,464,600 ±3,965,000	67.3 ±63.5	41.8 ±40.1	21.8 ±21.1	41 ±40	22.3 ±20.8	74.4 ±71.4
Côte d'Ivoire	4,243,600 ±3,384,600	67.7 ±57.7	44.4 ±37.8	28.9 ±24.1	50.1 ±42.2	37.3 ±31.1	46.1 ±41.3
Zimbabwe	4,149,900 ±4,142,800	45.5 ±51.3	19.7 ±22.2	8.2 ±9.2	16.6 ±18.6	5.7 ±6.7	100 ±112.3
Ghana	3,942,100 ±3,366,000	76.1 ±67.8	56.5 ±50	41.2 ±36.5	69.3 ±61.3	55.1 ±48.9	11.3 ±11
Liberia	3,442,400 ±1,384,000	83.7 ±34.5	67.9 ±28.2	51.5 ±21.7	85.4 ±35.6	68.2 ±29.4	1.2 ±0.6
Kenya	2,360,900 ±2,333,000	72.6 ±74.7	49 ±50.4	31.3 ±32.2	55.4 ±57	34.2 ±35.6	55 ±56.5
Senegal	1,934,700 ±1,947,000	47.8 ±52.8	24.6 ±26.1	11.6 ±12.2	22.1 ±23.4	6.3 ±7.4	100 ±112.5
Uganda	1,711,400 ±1,505,200	63 ±61.2	38.2 ±35.5	20.6 ±19.6	40.3 ±38.8	25.7 ±23.8	85.3 ±81.6
Chad	1,711,100 ±1,736,800	58.8 ±65.8	28 ±32.3	9 ±10.7	20.8 ±24.2	3.1 ±4	100 ±108.8
Equatorial Guinea	1,685,500 ±535,300	82.8 ±27.3	67.4 ±22.1	52.9 ±17.5	87 ±28.5	77.2 ±25.7	97.1 ±32.2
Malawi	1,552,200 ±1,430,200	64.7 ±63.7	38.7 ±38.6	21.1 ±21.2	39.5 ±39.3	17 ±17.1	100 ±97.7
Benin	1,481,700 ±1,472,700	50.3 ±57.3	19.7 ±22.2	6 ±7.2	14 ±16.2	2.6 ±2.9	100 ±109.2
Mali	959,900 ±977,000	16.8 ±24.5	1 ±1.6	0 ±0	0.2 ±0.4	0 ±0	100 ±125.2
Sudan	913,800 ±926,100	29.2 ±38.4	1.7 ±2.7	0 ±0	0 ±0	0 ±0	100 ±110.9
Guinea-Bissau	900,700 ±802,100	55.1 ±54.5	27.6 ±27.1	12.1 ±12	24.4 ±24.5	7.4 ±7.7	100 ±96.6
Togo	815,600 ±787,000	43 ±48.2	16.3 ±19	5.6 ±6.3	12.6 ±13.7	5.2 ±5.2	82.6 ±92.4
Sierra Leone	663,500 ±430,300	78.3 ±52.6	61.1 ±40.9	45.1 ±30.3	75 ±50.3	59.9 ±40.6	18.4 ±14
Botswana	356,200 ±360,000	83.2 ±82.9	63.9 ±63.7	7.7 ±8.7	35.7 ±36.8	0 ±0	100 ±99.5
South Africa	252,600 ±244,800	65 ±67.8	39.6 ±41.8	20.6 ±21.6	40.5 ±42.5	19.9 ±20.9	100 ±102.8
Burkina Faso	224,500 ±225,200	40.9 ±49.3	1.3 ±4.8	0 ±0	0 ±0	0 ±0	100 ±110.5
Namibia	211,500 ±216,000	10.5 ±19.8	4.9 ±5	0 ±0	0 ±2	0 ±0	100 ±127.9
Somalia	201,100 ±204,900	41.7 ±51.3	21 ±22.8	10.3 ±10.6	16.5 ±17	8.9 ±9.1	100 ±115.9
Burundi	135,400 ±120,100	62.1 ±57.7	38.1 ±35.1	12.2 ±11	28.5 ±26.4	4.2 ±3.7	98 ±90.7

Supplementary Table 1 (con't): Sensitivity analyses measuring the change in climate mitigation potential (CMP) associated with profitable areas. These account for leakage (10%, 20% and 30%), increase in development and maintenance cost (50% and 100%), and opportunity cost (from agricultural rents and timber production).

Country	Original analysis (CMP)	Leakage: 10% (% CMP)	Leakage: 20% (% CMP)	Leakage: 30% (% CMP)	Cost: +50% (% CMP)	Cost: +100% (% CMP)	Opportunity Cost (% CMP)
Gambia	85,900 ±85,200	70.2 ±72.1	40.6 ±42	16.2 ±17.2	33.5 ±35.6	3.8 ±4.8	100 ±101.5
Rwanda	52,300 ±44,600	74.2 ±63.7	55.8 ±47.2	26.8 ±23.5	60.4 ±52.4	15.4 ±13.9	98.5 ±85.1
Sao Tome and Principe	35,800 ±17,100	81.7 ±40.6	63.6 ±32.6	44 ±22.8	74 ±36.7	57 ±28.2	100 ±50.4
Comoros	6,000 ±3,600	0 ±13.6	0 ±0	0 ±0	0 ±0	0 ±0	100 ±87.4
Mayotte	5,900 ±3,300	35.2 ±24.1	16.5 ±13.5	0 ±4.2	22 ±18.5	0 ±0	100 ±68.7
**Asia-Pacific**	**581,832,200 ±311,789,600**	**85.5 ±46.3**	**70.8 ±38.5**	**55.7 ±30.5**	**91.2 ±49.7**	**80.2 ±44.4**	**42 ±23.4**
Indonesia	230,478,400 ±99,746,400	85.5 ±37.4	71.2 ±31.2	56.8 ±25	92.4 ±40.7	84.6 ±37.5	41.2 ±19
Malaysia	53,632,000 ±21,366,700	86.2 ±34.6	72.2 ±29.1	58 ±23.5	94.2 ±38.1	86.6 ±35.5	21.3 ±9.7
India	49,742,000 ±43,362,700	84.9 ±74.7	69.1 ±61.1	51.4 ±46.2	86.5 ±77.2	60.1 ±56	34.3 ±30.2
Thailand	39,054,400 ±26,658,300	85.7 ±59	71.2 ±49.1	56.3 ±39	92 ±63.6	81.6 ±57.1	27.3 ±18.8
Myanmar	35,182,200 ±21,480,500	83.7 ±52	66.9 ±41.9	49.7 ±31.4	83.1 ±52.4	65.9 ±42.1	10.2 ±6.8
Australia	33,746,100 ±23,335,000	85.6 ±59.7	70.2 ±49.3	54.3 ±38.4	89.6 ±63	73.9 ±53.3	96.8 ±67.3
Cambodia	28,307,400 ±17,178,600	87.2 ±53	74.3 ±45.2	61.2 ±37.3	98.4 ±60	94.1 ±57.9	73.6 ±44.7
China	28,294,400 ±16,089,800	86.1 ±49.3	72 ±41.3	57.6 ±33.2	93.6 ±53.9	85.4 ±49.6	32.9 ±20.6
Viet Nam	24,031,300 ±14,475,100	85.9 ±52.1	71.6 ±43.6	57 ±34.9	92.9 ±56.7	84.1 ±51.8	45.8 ±28.7
Laos	22,122,800 ±10,166,500	85.3 ±39.8	70.4 ±32.9	55.3 ±26.1	90.6 ±42.5	79.7 ±38	43.6 ±21
Papua New Guinea	16,503,600 ±6,223,400	74.2 ±30.7	51 ±21.4	30.8 ±13.3	55.5 ±23.8	28.9 ±13.2	24.9 ±10
Philippines	10,133,500 ±5,156,000	84.2 ±43.7	68.2 ±35.6	51.3 ±27.1	85.4 ±45	67.9 ±36.7	62.3 ±32.3
Bangladesh	4,154,400 ±2,934,100	86.2 ±61.1	72.4 ±51.4	58.6 ±41.7	94.8 ±67.4	87.8 ±63	77.6 ±55
Sri Lanka	4,153,800 ±2,540,800	86.8 ±53.3	73.5 ±45.2	60.3 ±37.1	97.1 ±59.7	91.7 ±57.2	100 ±61.3
Brunei Darussalam	1,101,000 ±431,600	85.3 ±33.9	70.5 ±28.2	54.8 ±22.2	90.5 ±36.4	77.8 ±31.9	48.4 ±20.9
Taiwan	598,200 ±308,400	83.4 ±44.1	65.9 ±35.1	48.6 ±26.1	81.7 ±43.9	63.8 ±35.3	100 ±52.6
Timor-Leste	471,000 ±261,300	84.9 ±47.7	69.6 ±39.4	53.6 ±30.6	88.8 ±50.5	74.6 ±43.1	100 ±56
Hong Kong	124,600 ±73,300	84.6 ±50.9	66.7 ±40.6	49.5 ±30.3	82 ±49.7	60.8 ±38.4	93.7 ±55.8
Singapore	1,200 ±1,000	87.5 ±73.7	75 ±63.2	62.5 ±52.6	100 ±84.2	100 ±84.2	58.7 ±49.4

Supplementary Table 2: Comparisons between volumes of estimated investible carbon and verified carbon units (VCUs) issued within verified tropical VCS project areas between 2005–2018. Data obtained from https://verra.org/.

Country	ID	Name	Status	# Years with data	Years	VCUs ($MtCO_2$ yr^{-1})	Estimated Values ($MtCO_2$ yr^{-1})
Brazil	963	The Purus Project	Verified, under verification	7	2011–2017	0.0095 ± 0.0051	0.0374 ± 0.0011
	977	RMDLT Portel-Para REDD Project	Verification approved	5	2009–2017	0.097 ± 0.113	0.1748 ± 0.0053
	1112	The Russas Project	Verified, under verification	2	2011–2014	0.0078 ± 0.0024	0.0422 ± 0.0013
	1113	The Valparaiso Project	Verified, under verification	3	2012–2014	0.0032 ± 0.0021	0.0228 ± 0.0007
	1382	The Envira Amazonia Project - A Tropical Forest Conservation Project in Acre, Brazil	Verified, under verification	4	2012–2018	0.0317 ± 0.0374	0.0597 ± 0.0018
Cambodia	1650	Reduced Emissions from Deforestation and Degradation in Keo Seima Wildlife Sanctuary	Verification approved	3	2013–2017	0.1182 ± 0.1575	0.8828 ± 0.0266
Colombia	856	Chocó-Darién Conservation Corridor REDD Project	Verification approved	2	2010–2012	0.0025 ± 0.002	0.0038 ± 0.0001
DR Congo	934	Mai Ndombe REDD+ Project	Verification approved	6	2011–2016	0.6565 ± 0.58	0.4573 ± 0.0137
	1359	Isangi REDD+ Project	Verification approved	5	2009–2013	0.0219 ± 0.0243	0.0807 ± 0.0024
Ethiopia	1340	Bale Mountains Eco-region REDD+ Project	Verification approved	4	2012–2015	0.7359 ± 0.5087	1.1743 ± 0.034
Guatemala	1622	REDD+ Project for Caribbean Guatemala: The Conservation Coast	Verified, under verification	7	2012–2018	0.1476 ± 0.1042	0.1899 ± 0.0056
Indonesia	674	Rimba Raya Biodiversity Reserve Project	Verification approved	9	2009–2017	0.1528 ± 0.1728	0.159 ± 0.0035
Kenya	1408	Chyulu Hills REDD+ Project	Verification approved	3	2013–2016	0.1056 ± 0.1089	0.0227 ± 0.0003
Madagascar	1215	The Makira Forest Protected Area in Madagascar	Verification approved	2	2005–2013	0.0026 ± 0.0018	0.0634 ± 0.0016
Malawi	1168	Kulera Landscape REDD+ Program for Co-Managed Protected Areas, Malawi	Verification approved	1	2009–2009	0.0025	0.1765 ± 0.0047
Paraguay	1403	The Paraguay Forest Conservation Project	Verification approved	1	2010–2010	0.0006	0.0018 ± 0.0001

Supplementary Table 2 (con't): Comparisons between volumes of estimated investible carbon and verified carbon units (VCUs) issued within verified tropical VCS project areas between 2005–2018. Data obtained from https://verra.org/.

Country	ID	Name	Status	# Years with data	Years	VCUs ($MtCO_2$ yr^{-1})	Estimated Values ($MtCO_2$ yr^{-1})
Peru	844	Madre de Dios Amazon REDD Project	Verification approved	7	2009–2018	0.0174 ± 0.0172	0.0951 ± 0.0028
	944	Alto Mayo Conservation Initiative	Verified, under verification	4	2008–2016	0.0472 ± 0.0396	0.0243 ± 0.0007
	958	Biocorredor Martin Sagrado Project	Verification approved	1	2010–2010	0.005	0.0469 ± 0.0014
	985	Cordillera Azul National Park REDD Project	Verification approved	7	2008–2017	0.4019 ± 0.3269	0.0001 ± 0
	1360	Forest Management to Reduce Deforestation and Degradation in Shipibo Conibo and Cacataibo Indigenous Communities of Ucayali Region	Verification approved	8	2010–2017	0.1426 ± 0.1762	0.1184 ± 0.0033
Sierra Leone	1201	Gola REDD Project	Verification approved	3	2012–2014	0.0135 ± 0.0122	0.08 ± 0.0024
Tanzania	1325	Mjumita community forest project (Lindi)	Verification approved	1	2012–2012	0.0004	0.0596 ± 0.0015
Zambia	1202	Lower Zambezi REDD+ Project	Verification approved	10	2009–2018	0.0098 ± 0.0071	0.0251 ± 0.0006
Zimbabwe	902	Kariba REDD+ Project	Verification approved	6	2011–2016	0.0327 ± 0.033	0.0815 ± 0.002

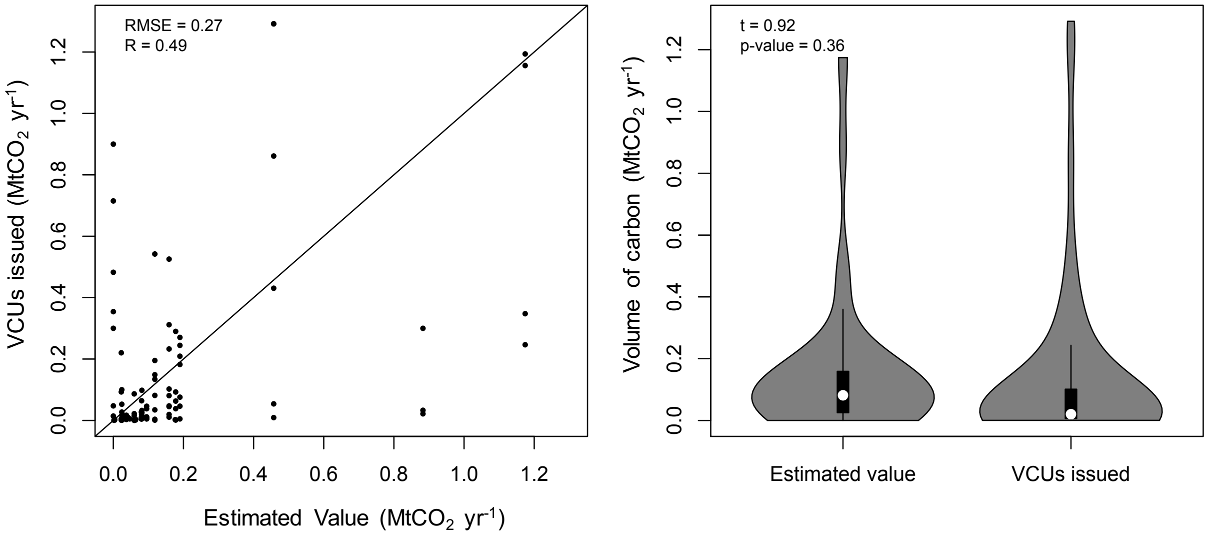

Supplementary Figure 1: Estimates of investible carbon credits are significantly correlated to the verified credits generated from existing verified tropical avoided deforestation projects. Data from Supplementary Table 1.

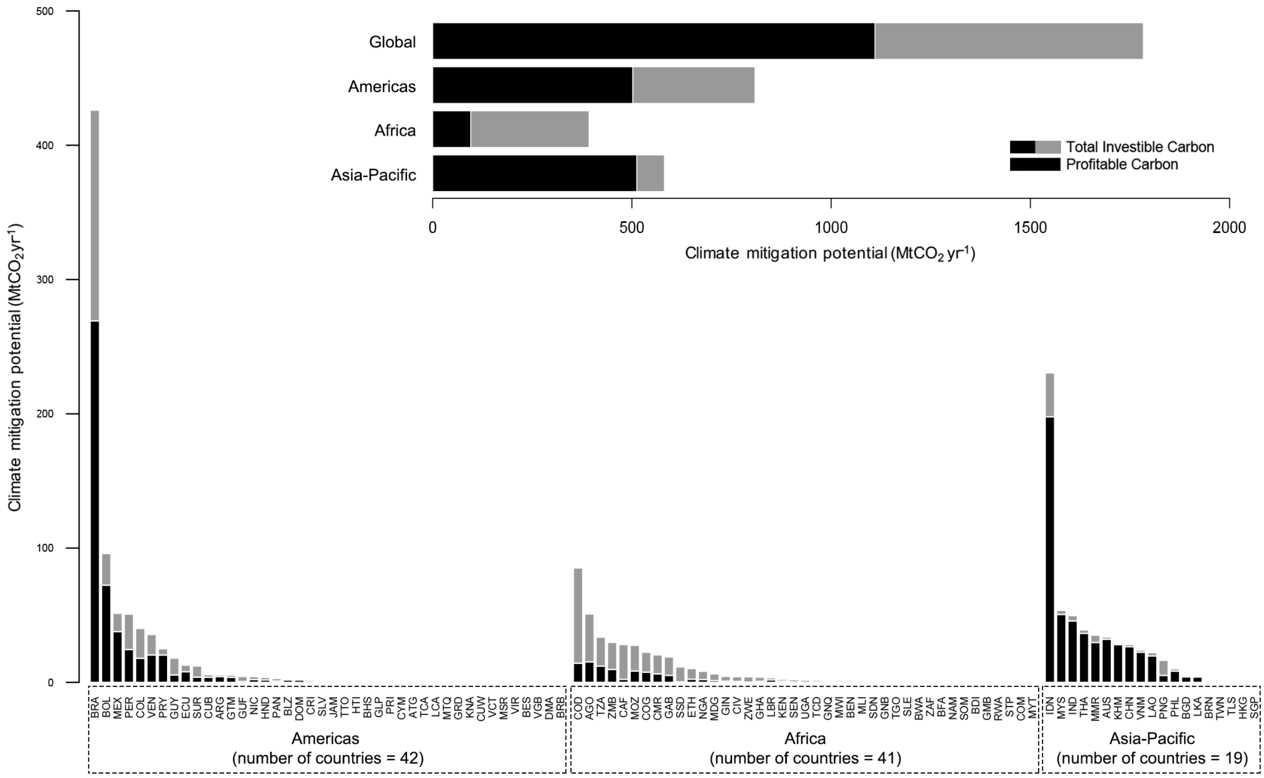

Supplementary Figure 2: Difference between volumes of investible and profitable carbon credit at global, regional and country levels. Country abbreviations are based on ISO 3166-1 alpha-3 codes.
