# Supplementary material for: Carbon prospecting in tropical forests for climate change mitigation
Source: Nat Commun. 2021 Feb 24;12:1271. doi: 10.1038/s41467-021-21560-2 (PMC7904812; doi:10.1038/s41467-021-21560-2)
Supplement: Supplementary file 2 — Reporting Summary [file 41467_2021_21560_MOESM2_ESM.pdf]

nature research

Corresponding author(s): Lian Pin Koh; Yiwen Zeng

Last updated by author(s): Jan 28, 2021

Reporting Summary

Nature Research wishes to improve the reproducibility of the work that we publish. This form provides structure for consistency and transparency in reporting. For further information on Nature Research policies, see Authors & Referees and the Editorial Policy Checklist.

Statistics

For all statistical analyses, confirm that the following items are present in the figure legend, table legend, main text, or Methods section.

n/a	Confirmed	
☐	☒	The exact sample size (*n*) for each experimental group/condition, given as a discrete number and unit of measurement
☐	☒	A statement on whether measurements were taken from distinct samples or whether the same sample was measured repeatedly
☐	☒	The statistical test(s) used AND whether they are one- or two-sided<br>*Only common tests should be described solely by name; describe more complex techniques in the Methods section.*
☐	☒	A description of all covariates tested
☐	☒	A description of any assumptions or corrections, such as tests of normality and adjustment for multiple comparisons
☐	☒	A full description of the statistical parameters including central tendency (e.g. means) or other basic estimates (e.g. regression coefficient) AND variation (e.g. standard deviation) or associated estimates of uncertainty (e.g. confidence intervals)
☒	☐	For null hypothesis testing, the test statistic (e.g. *F*, *t*, *r*) with confidence intervals, effect sizes, degrees of freedom and *P* value noted<br>*Give P values as exact values whenever suitable.*
☒	☐	For Bayesian analysis, information on the choice of priors and Markov chain Monte Carlo settings
☒	☐	For hierarchical and complex designs, identification of the appropriate level for tests and full reporting of outcomes
☒	☐	Estimates of effect sizes (e.g. Cohen's *d*, Pearson's *r*), indicating how they were calculated

Our web collection on statistics for biologists contains articles on many of the points above.

Software and code

Policy information about availability of computer code

Data collection	Primary data was not collected in this study. Instead, we utilize a range raster layers to estimate climate mitigation potential and return on investments. We also utilize data from the Verra database to validate our estimates. These are listed in the Methods.
Data analysis	All analyses was performed in R version 3.6.0, utilizing the package "raster" for processing and calculations of raster layers. Map visualizations were formed in QGIS.

For manuscripts utilizing custom algorithms or software that are central to the research but not yet described in published literature, software must be made available to editors/reviewers. We strongly encourage code deposition in a community repository (e.g. GitHub). See the Nature Research guidelines for submitting code & software for further information.

Data

Policy information about availability of data

All manuscripts must include a data availability statement. This statement should provide the following information, where applicable:

- Accession codes, unique identifiers, or web links for publicly available datasets
- A list of figures that have associated raw data
- A description of any restrictions on data availability

All maps generated are available from Zenodo, DOI: 10.5281/zenodo.4287780

Field-specific reporting

Please select the one below that is the best fit for your research. If you are not sure, read the appropriate sections before making your selection.

☐ Life sciences ☐ Behavioural & social sciences ☒ Ecological, evolutionary & environmental sciences

Ecological, evolutionary & environmental sciences study design

All studies must disclose on these points even when the disclosure is negative.

Study description	Investing in forest protection is a way to generate tradable carbon (C) credits to support biodiversity conservation and climate change mitigation. Here the authors assess and map the global supply of tropical forest C credits with the goal of informing climate policy and investments.
Research sample	Not applicable
Sampling strategy	Not applicable
Data collection	Data was collected from multiple publication and online-datasets by YZ and TVS.
Timing and spatial scale	Analyses was done for the global tropics, based on data collected between 2012–2017.
Data exclusions	No data was excluded
Reproducibility	We performed uncertainty analyses to ensure reproducibility
Randomization	No randomization was needed for this study.
Blinding	No blinding was needed for this study.

Did the study involve field work? ☐ Yes ☒ No

Reporting for specific materials, systems and methods

We require information from authors about some types of materials, experimental systems and methods used in many studies. Here, indicate whether each material, system or method listed is relevant to your study. If you are not sure if a list item applies to your research, read the appropriate section before selecting a response.

Materials & experimental systems

n/a	Involved in the study
☒	☐ Antibodies
☒	☐ Eukaryotic cell lines
☒	☐ Palaeontology
☒	☐ Animals and other organisms
☒	☐ Human research participants
☒	☐ Clinical data

Methods

n/a	Involved in the study
☒	☐ ChIP-seq
☒	☐ Flow cytometry
☒	☐ MRI-based neuroimaging
